# GOYA: Leveraging Generative Art for Content-Style Disentanglement †

**DOI:** 10.3390/jimaging10070156

**Published:** 2024-06-26

**Authors:** Yankun Wu, Yuta Nakashima, Noa Garcia

**Affiliations:** Intelligence and Sensing Lab, Osaka University, Suita 565-0871, Osaka, Japan; n-yuta@ids.osaka-u.ac.jp (Y.N.); noagarcia@ids.osaka-u.ac.jp (N.G.)

**Keywords:** art analysis, representation disentanglement, text-to-image generation

## Abstract

The content-style duality is a fundamental element in art. These two dimensions can be easily differentiated by humans: content refers to the objects and concepts in an artwork, and style to the way it looks. Yet, we have not found a way to fully capture this duality with visual representations. While style transfer captures the visual appearance of a single artwork, it fails to generalize to larger sets. Similarly, supervised classification-based methods are impractical since the perception of style lies on a spectrum and not on categorical labels. We thus present *GOYA*, which captures the artistic knowledge of a cutting-edge generative model for disentangling content and style in art. Experiments show that *GOYA* explicitly learns to represent the two artistic dimensions (content and style) of the original artistic image, paving the way for leveraging generative models in art analysis.

## 1. Introduction

Content and style are two fundamental elements in the analysis of art. Content refers to the subject matter depicted in the artwork, answering the question of *what scene the artwork depicts*, e.g., a girl chasing a butterfly, fruits on a table, or a street scene near a river. On the other hand, style corresponds to *how the artwork looks*, focusing on the visual appearance of the image, such as color compositions, brushstrokes, and perspective. Each artwork is characterized by a distinctive integration of content and style, making the disentanglement of these two elements an essential aspect of the study of digital humanities.

While humans can easily distinguish content and style, from a computer vision perspective, the boundary between content and style is not so clear. Generally, in the computer vision field, object detection techniques are widely applied to analyzing content in artworks [1]. However, artworks may contain similar objects while still conveying different *subject matters*. Similarly, the automatic analysis of style presents its own challenges. Without a formal definition of what visual appearance is, there is a degree of vagueness and subjectivity in the computation of style. Some methods [2,3] classify style by relying on well-established attributes, such as author or artistic movement. While this approach may work on certain applications, such as artist identification [4], it may not be applicable to other tasks such as style transfer [5] or image search [6]. In style transfer, for example, style is defined as the low-level features of an image (e.g., colors, brushstrokes, shapes). However, in a broader sense, style is not formed by a single image but by a set of artworks that share a common visual appearance [7].

To address these challenges, most methods for art analysis rely on full supervision [3,8], requiring corresponding content or style labels for each image in the dataset. Although some art datasets with labeled attributes are available (e.g., WikiArt [9], The Met [6], APOLO [10]), additional issues arise. Firstly, the attributes of new artworks still require experts to annotate them. Moreover, the annotated labels commonly are words describing general traits of artwork collections, making it difficult to convey subtle differences between artworks. For instance, what scene does a painting in the *still life* genre depict? What does the visual appearance of an *Expressionism* style painting look like? While we can infer some of the common attributes they may carry, e.g., inanimate subjects in the *still life* painting and strong subjective emotions in the *Expressionism* painting, detailed attributes such as depicted concepts, color composition, and brushstrokes still remain unknown. When training based on labels, it is challenging to capture the subtle content and style discrepancies in images. To resolve this problem, some work [11] leverages natural language descriptions instead of categorical classes. Although natural language can overcome the ambiguity and rigidity of labels, they still require human experts to write descriptions for each image.

In our work, we exploit the generative power of a popular text-to-image model, Stable Diffusion [12], and propose leveraging the distilled knowledge as a prior to learn disentangled content and style embeddings of paintings. Given a prompt specifying the desired content and style, Stable Diffusion can generate a diverse set of synthetic images while maintaining consistency with the prompt. The subtle characteristics of content and style in the synthetically generated images can be controlled through well-defined prompts. Thus, free from direct human annotations, we train on the generated images to disentangle content and style using contrastive learning. Previous work also shows that Stable Diffusion generated images can be useful for image classification [13].

**Figure 1:**
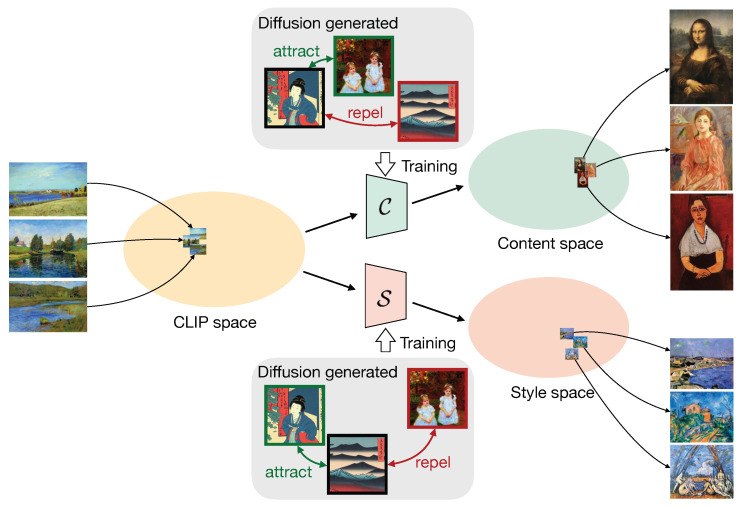
An overview of our method, GOYA. By using Stable Diffusion generated images, we disentangle content and style spaces from CLIP space, where content space represents semantic concepts and style space captures visual appearance.

The intuition behind our method, named GOYA (disentanGlement of cOntent and stYle with generAtions), is that, although there is no explicit boundary between different contents or styles, significant dissimilarities can be distinguished by comparison. Our simple yet effective model (Figure 1) first extracts joint content-style embeddings using a pre-trained Contrastive Language-Image Pretraining (CLIP) image encoder [14], and then applies two independent transformation networks to learn disentangled content and style embeddings. These transformation networks are trained on the generated synthetic images with contrastive learning, reducing the reliance on human image-level annotations.

We conducted three tasks and an ablation study on a popular benchmark of paintings, the WikiArt dataset [9]. We show that, even with distilled knowledge from Stable Diffusion, our model achieves better disentanglement between content and style compared to other models trained on real paintings. Additionally, experiments demonstrate that the resulting disentangled spaces are useful for downstream tasks such as similarity retrieval and art classification. In summary, our contributions are as follows:

- We design a disentanglement model to obtain disentangled content and style space derived from CLIP’s latent space.
- We train our model with synthetic images rather than real paintings, leveraging the capabilities of Stable Diffusion and prompt design.
- Results indicate that the knowledge in Stable Diffusion can be effectively distilled for art analysis, performing well in content-style disentanglement, art retrieval, and art classification.

Our findings pave the way for the adoption of generative models in digital humanities, not only for generation but also for analysis. The code is available at https://github.com/yankungou/GOYA (accessed on 18 April 2024).

## 2. Related Work

### 2.1. Art Analysis

The use of computer vision techniques for art analysis has been an active research topic for decades, particularly in tasks such as attribute classification [15,16], object recognition [17,18], and image retrieval [1,6]. Fully-supervised tasks (e.g., genre or artist classification [15]) have achieved outstanding results by leveraging neural networks trained on annotated datasets [19,20]. However, image annotations have some limitations, particularly in the categorization of styles. Multiple datasets [21,22,23,24] provide style labels, which abundant research [4,25,26,27] has utilized for style classification. This direction of work assumes style to be a static attribute rather than dynamic and evolving [7]. A different interpretation is provided by style transfer [5] where a model extracts the low-level representation of a *stylized image* (e.g., a painting) and applies it to a *content image* (e.g., a plain photograph), defining style based on a single artwork’s characteristics like color, shape, and brushstroke. To address the limitations of rigid labels in supervised learning and the narrow focus on a single image in style transfer, we propose learning disentangled embeddings of content and style through similarity comparisons leveraging the flexibility of a text-to-image generative model.

### 2.2. Representation Disentanglement

Disentangling representation plays an essential role in various computer vision tasks such as style transfer [28,29], image manipulation [30,31], and image-to-image translation [32,33]. The goal is to discover discrete factors of variation in data, thus improving the interpretability of representations and enabling a wide range of downstream applications. Previous work on disentangling attributes like azimuth, age, or gender has utilized adversarial learning [34] or variational autoencoders [35], aiming to encourage discrete properties in a single latent space. For content and style disentanglement, approaches apply generative models [28], a diffusion model [36], or an autoencoder architecture with contrastive learning [37]. In the art domain, ALADIN [37] concatenates the adaptive instance normalization (AdaIN) [38] feature into the style encoder to learn style embedding for visual searching. Kotovenko et al. [28] propose fixpoint triplet loss and disentanglement loss for performing better style transfer. However, these approaches lack semantic analysis of content embeddings in paintings. Recently, Vision Transformer (ViT)-based models has shown the ability to obtain structure and appearance embeddings [39,40]. DiffuseIT [36] and Splice [39] learn content and style embeddings by utilizing the keys and the global [CLS] token of pre-trained DINO [40]. In our work, taking advantage of the generative model, our approach builds a simple framework to decompose the latent space into content and style spaces with contrastive learning, exploring the use of generated images in representation learning.

### 2.3. Text-to-Image Generation

Text-to-image generation models aim to produce synthetic images based on given text inputs. Fueled by datasets containing vast text-image pairs that have emerged in recent years, numerous text-to-image generation models have been developed [12,41,42]. For instance, CogView [42] is trained on 30 million text-image pairs, while DALL-E 2 [41] is trained on 650 million text-image pairs. One of the main challenges faced by these models is achieving semantic coherence between guiding texts and generated images. This challenge has been addressed by using pre-trained CLIP embeddings [14] to construct aligned text and image features in the latent space [43,44,45]. Another challenge is obtaining high-resolution synthetic images. GAN-based models [46,47] have shown good performance in improving the quality of generated images; however, they suffer from instability during training. Leveraging the superior training stability, approaches based on diffusion models [12] have recently emerged as a popular tool for generating near-human quality images. Despite the rapid development of models for image generation, how the features of synthetic images can be utilized remains an underexplored area of research. In this paper, we study the potential of generated images for enhancing representation learning.

### 2.4. Training on Synthetic Images

With the increasing availability of open-sourced applications in generative models, synthetic images can be collected and integrated into training data, potentially impacting the development and performance of future models [48]. Several studies have investigated the impact of synthetic images across various aspects, including art forgeries [49], learnt representations [50], datasets [51], model training [52,53], and classification [13,51]. Tian et al. [50] demonstrate that training solely on synthetic images using self-supervised methods can yield better representations than training on real images of the same sample size. Sariyildiz et al. [13] show that models trained on synthetic ImageNet clones achieve comparable performance on classification tasks to those trained on real image. Azizi et al. [52] demonstrate that augmenting real data with generated images during training improves classification accuracy score (CAS) [54]. In the art domain, Ostmeyer et al. [49] find that training with synthetic images enhances the recognition of human-made art forgeries. In our work, we explore leveraging synthetic images for content and style disentanglement in art paintings.

## 3. Preliminaries

### 3.1. Stable Diffusion

Diffusion models [12,55] are generative methods trained in two stages: a forward process with a Markov chain to transform input data to noise, and a reversed process to reconstruct data from the noise, achieving high-quality performance in image generation.

To reduce training costs and accelerate the inference process, Stable Diffusion [12] trains the diffusion process in the latent space instead of the pixel space. Given a text prompt as input condition, the text encoder transforms the prompt to a text embedding. Then, by feeding the embedding into the UNet through a cross-attention mechanism, the reversed diffusion process generates an image embedding in the latent space. Finally, the image embedding is fed to the decoder to generate a synthetic image.

In this work, we define symbols as follows: given a text prompt $x=\{x^{C},x^{S}\}$ as input, we can obtain the generated image *y*. The text $x^{C}$ represents content description and $x^{S}$ denotes style description, where {·} indicates a comma-separated string concatenation.

### 3.2. CLIP

CLIP [14] is a text-image matching model that aligns text and image embeddings in the same latent space. It shows high consistency between the visual concepts in the image and the semantic concepts in the corresponding text. The text encoder $E_{T}$ and image encoder $E_{I}$ of CLIP are trained with 440 million text-image pairs, showing outstanding performance on various text and image downstream tasks, such as zero-shot prediction [56,57] and image manipulation [44,45,58]. Given the text *x* and an image *y*, the CLIP embeddings *f* from text, and *g* from image, both in $R^{d}$, can be computed as follows:

(1) $$\begin{array}{cc} f & =E_{T}\left(x\right), \end{array}$$

(2) $$\begin{array}{cc} g & =E_{I}\left(y\right). \end{array}$$

To exploit the multi-modal CLIP space, we employ the pre-trained CLIP image encoder $E_{I}$ to obtain CLIP image embeddings as the prerequisite for the subsequent disentanglement model. Moreover, during the training stage, the CLIP text embedding of a prompt is applied to acquire the semantic concepts of the generated image.

## 4. GOYA

We aim to learn the disentangled content and style embeddings of artworks in two different spaces. To collect a diverse set of artistic images with various content and style, we leverage Stable Diffusion to generate synthetic images based on specific content and style descriptions. By training with contrastive loss, our GOYA model effectively learns the proximity of different artworks in two spaces, guided by text prompts.

**Figure 2:**
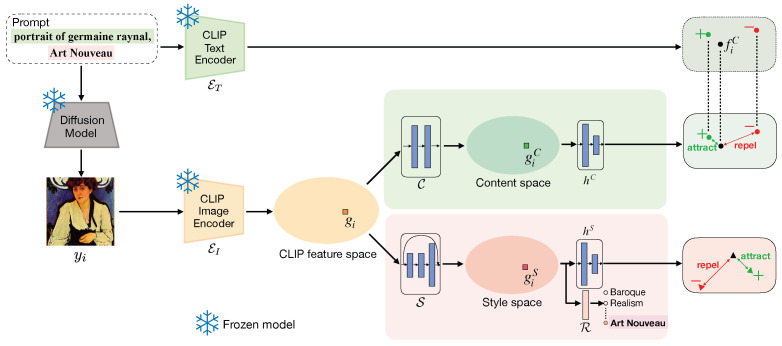
Details of our proposed method, GOYA, for content and style disentanglement. Given a synthetic prompt containing content (first part of the prompt, in green) and style (second part of the prompt, in red) descriptions, we generate synthetic diffusion images. We compute CLIP embeddings with the frozen CLIP image encoder, and generate content and style disentangled embeddings with two dedicated encoders C and S, respectively. In the training stage, projectors $h^{C}$ and $h^{S}$ and style classifier R are used to train GOYA with contrastive learning. For content, contrastive learning pairs are chosen based on the text embedding of content description in the prompt extracted by frozen CLIP text encoder. For style, contrastive learning pairs are chosen based on the style description in the prompt.

Figure 2 shows an overview of GOYA. Given a mini-batch of *N* prompts ${\left\{x_{i}\right\}}_{i=0}^{N}$, where $x_{i}=\left\{x_{i}^{C},x_{i}^{S}\right\}$ with comma-connected content and style descriptions, we obtain diffusion generated images $y_{i}$ using Stable Diffusion. We then compute CLIP image embeddings $g_{i}$ by Equation (2) and use a content and a *style encoder* to obtain disentangled content and style embeddings in two different spaces, respectively. As previous research has shown [59] that content and style possess different properties, while content embeddings correspond to higher layers in the deep neural network and style embeddings correspond to lower layers. Accordingly, we design an asymmetric network architecture for extracting content and style, a common approach in the art analysis domain [20,28,37,59].

### 4.1. Content Encoder

The content encoder C maps CLIP image embedding $g_{i}$ to content embedding $g_{i}^{C}$ as follows:

(3) $$g_{i}^{C}=C\left(g_{i}\right),$$

C is a two-layer perceptron (MLP) with ReLU non-linearity. Following previous research [60], to make content $g_{i}^{C}$ highly linear, during training, we add a non-linear projector $h^{C}$ on top of the content encoder, which is a three-layer MLP with ReLU non-linearity.

### 4.2. Style Encoder

Style encoder S also maps CLIP image embedding $g_{i}$ but to style embedding $g_{i}^{S}$ as follows:

(4) $$g_{i}^{S}=S\left(g_{i}\right).$$

S is a three-layer MLP with ReLU non-linearity. In particular, following [61], we apply a skip connection before the last ReLU non-linearity in S. Similar to the content encoder, non-linear projector $h^{S}$ with the same structure as $h^{C}$ is added after S to facilitate contrastive learning.

### 4.3. Content Contrastive Loss

Unlike prior research [28], which defines content similarity only solely based on style-transferred images originating from the same source, we use a broader definition of content similarity. We introduce a soft-positive selection strategy that identifies pairs of images with similar content according to their semantic similarity. That is, two images sharing similar semantic concepts are designated as a positive pair, whereas images lacking semantic similarity are considered negative pairs.

To quantify *semantic similarity* between a pair of images, we exploit the CLIP latent space and conduct text similarity between the associated texts. Given the content description $x_{i}^{C}$ of the image $y_{i}$, we consider the CLIP text embedding $f_{i}^{C}=E_{T}\left(x_{i}^{C}\right)$ as a proxy for the content of $y_{i}$. Therefore, for a pair of two diffusion images $\left(y_{i},y_{j}\right)$ and a text similarity threshold $\epsilon^{T}$, they are considered a positive pair if $D_{ij}^{T}\leq \epsilon^{T}$, where $D_{ij}^{T}$ is the text similarity obtained by the cosine distance between the CLIP text embedding $f_{i}^{C}$ and $f_{j}^{C}$. The content contrastive loss is defined as follows:

(5) $$L_{ij}^{C}=1_{\left[D_{ij}^{T}\leq \epsilon^{T}\right]}\left(1-D_{ij}^{C}\right)+1_{\left[D_{ij}^{T}>\epsilon^{T}\right]}\max\left(0,D_{ij}^{C}-\epsilon_{c}\right),$$

where $1_{\left[\cdot \right]}$ is the indicator function that yields 1 when the condition is true and 0 otherwise. $D_{ij}^{C}$ is the cosine distance between $h_{C}\left(g_{i}^{C}\right)$ and $h_{C}\left(g_{j}^{C}\right)$, which are the content embeddings of images after projection. $\epsilon_{c}$ is the margin that constrains the minimum distance of negative pairs.

### 4.4. Style Contrastive Loss

The style contrastive loss is defined based on the style description $x^{S}$ given in the input prompt. If a pair of images share the same style class, then they are considered a positive pair, indicating that their style embeddings should be close in the style space. Otherwise, they are deemed a negative pair, and they should be pushed away from each other. Given $\left(y_{i},y_{j}\right)$, the style contrastive loss can be computed as follows:

(6) $$L_{ij}^{S}=1_{\left[x_{i}^{S}=x_{j}^{S}\right]}\left(1-D_{ij}^{S}\right)+1_{\left[x_{i}^{S}\neq x_{j}^{S}\right]}\max\left(0,D_{ij}^{S}-\epsilon^{S}\right),$$

where $D_{ij}^{S}$ is the cosine distance between the style embeddings $h^{S}\left(g_{i}^{S}\right)$ and $h^{S}\left(g_{j}^{S}\right)$ after projection, and $\epsilon^{S}$ is the margin.

### 4.5. Style Classification Loss

To learn the general attributes of each style, we introduce a style classifier R to predict the style description (given as $x_{i}^{S}$) based on the embedding $g_{i}^{S}$ of image $y_{i}$. Prediction $w_{i}^{S}$ by the classifier is given by

(7) $$w_{i}^{S}=R\left(g_{i}^{S}\right),$$

where R is a linear layer network. For training, we use softmax cross-entropy loss, which is denoted by $L_{i}^{SC}$. Note that the training of this classifier does not rely on human annotations, but on the synthetic prompts and generated images by Stable Diffusion.

### 4.6. Total Loss

In the training process, we compute the sum of three losses. The overall loss function in a mini-batch is formulated as

(8) $$L=\lambda^{C}\sum_{ij}L_{ij}^{C}+\lambda^{S}\sum_{ij}L_{ij}^{S}+\lambda^{SC}\sum_{i}L_{i}^{SC},$$

where $\lambda^{C}$, $\lambda^{S}$, and $\lambda^{CS}$ are parameters to control the contributions of losses. We set $\lambda^{C}=\lambda^{S}=\lambda^{CS}=1$. The summations over *i* and *j* are computed for all pairs of images in the mini-batch, and the summation over *i* is for all images in the mini-batch.

## 5. Evaluation

We evaluate GOYA on three tasks: disentanglement (Section 5.5), classification (Section 5.7), and similarity retrieval (Section 5.6). We also conduct an ablation study in Section 5.8.

### 5.1. Evaluation Data

To assess content and style in the classification task, we utilize genre and style movement labels in art datasets that can be served as substitutes for presenting content and style, even if they do not entirely satisfy our definitions in this paper. In detail, the genre labels indicate the type of scene depicted in the paintings, such as “portrait” or “cityscape”, while style movement labels correspond to artistic movements such as “Impressionism” and “Expressionism”. We use the WikiArt dataset [9] for evaluation, a popular artwork dataset with both genre and style movement annotations. The dataset comprises a total of 81,445 paintings: 57,025 in the training set, 12,210 in the validation set, and 12,210 in the test set, with three types of labels: 23 artists, 10 genres, and 27 style movements. All evaluation results are computed on the test set.

### 5.2. Training Data

Baselines reported on WikiArt are typically trained with the WikiArt training set. GOYA is trained with generated images by Stable Diffusion, which are described in the next paragraph. Additionally, the training dataset of Stable Diffusion LAION-5B [62] contains over five billion image–text pairs, which contain some paintings from the WikiArt test set. We examine other models trained on generated images, which are equally affected by this issue.

### 5.3. Image Generation Details

To generate images resembling human-made paintings, we relied on craft prompts $x=\{x^{C},x^{S}\}$ as explained in Section 3.1. For simplicity, we selected titles of paintings as $x^{C}$ and style movements as $x^{S}$, although alternative definitions of content and style descriptions could be used. In total, there are 43,610 content descriptions $x^{C}$, and 27 style descriptions $x^{S}$. For each $x^{C}$, we randomly selected five $x^{S}$ to generate five prompts *x*. Then, each prompt generated five images with random seeds. In total, we obtained 218,050 prompts and 1,090,250 synthetic images. We split the generated images into 981,225 training and 109,025 validation images. We used Stable Diffusion v1.4 (https://github.com/CompVis/stable-diffusion, accessed on 1 September 2022) and generated images of size 512×512 through 50 PLMS [63] sampling steps.

**Figure 3:**
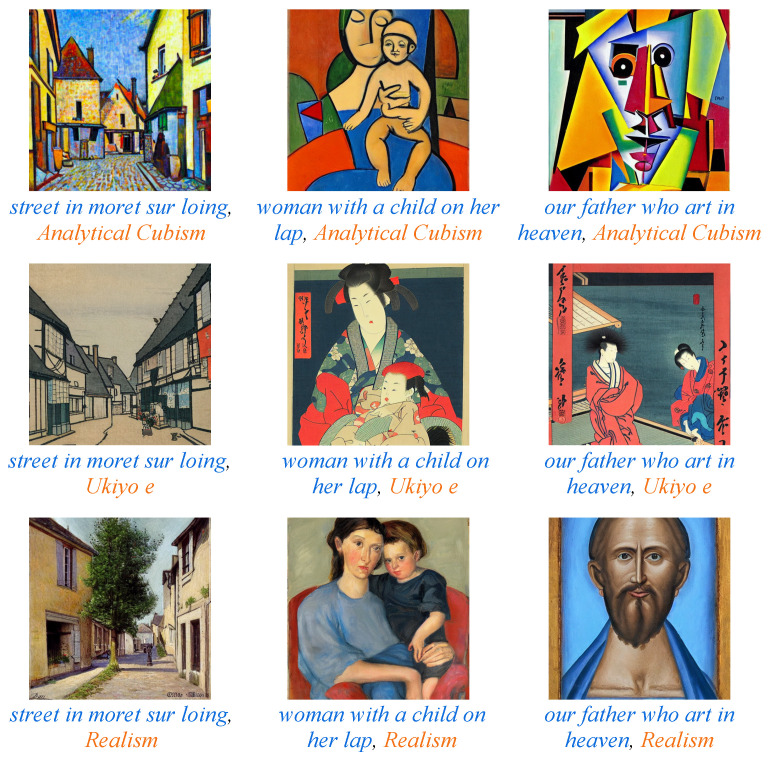
Examples of prompts and the corresponding generated diffusion images. The first part of the prompt (in blue) denotes the content description $x^{C}$, and the second part (in orange) is the style description $x^{S}$. Each column depicts the same content $x^{C}$ while each row depicts one style $x^{S}$.

Figure 3 depicts examples of diffusion generated images created by the specified prompts. We observed that the depicted scene is consistent with the content description in the prompts. Images in the same column have the same $x^{C}$ but different $x^{S}$, exhibiting a high level of agreement in content while carrying significant differences in style. Likewise, images in the same row have the same $x^{S}$ but different $x^{C}$, and paint different scenes or objects while maintaining a similar style. However, some content descriptions are religious, such as $x^{C}$ in the third column, “our father who art in heaven”. In such cases, achieving agreement on the semantic consistency between the generated images and the prompts may pose challenges.

### 5.4. GOYA Details

For the CLIP image and text encoders, we employ the pre-trained weights of CLIP-ViT-B/32 models (https://github.com/openai/CLIP, accessed on 1 October 2022). The margin for computing contrastive losses is set to $\epsilon^{C}=\epsilon^{S}=0.5$. In the indicator function for the content contrastive loss, the threshold $\epsilon^{T}$ is set to 0.25. We use the Adam optimizer [64] where base learning rate =0.0005 and decay rate =0.9. GOYA is trained on four A6000 GPUs with Distributed Data Parallel in PyTorch (https://pytorch.org/, accessed on 1 October 2022). In each device, the batch size is set as 512. Before being fed into CLIP, images are resized to 224×224 pixels. The architectural details of GOYA are shown in Table 1.

### 5.5. Disentanglement Evaluation

To measure content and style disentanglement quantitatively, we compute the distance correlation (DC) [65] between content and style embeddings, which is specially designed for content and style disentanglement evaluation. Let $G^{C}$ and $G^{S}$ denote matrices containing all content and style embeddings in the WikiArt test set, i.e., $G^{C}=\left(g_{1}^{C}\;\;\cdots \;\;g_{N}^{C}\right)$ and $G^{S}=\left(g_{1}^{S}\cdots \;\;g_{N}^{S}\right)$. For an arbitrary pair (i,j) of embeddings, the distances $p_{ij}^{C}$ and $q_{ij}^{S}$ can be computed by

(9) $$p_{ij}^{C}=∥g_{i}^{C}-g_{j}^{C}∥,\;\;\;p_{ij}^{S}=∥g_{i}^{S}-g_{j}^{S}∥,$$

where ∥·∥ gives the Euclidean distance. Let ${\bar{p}}_{i\cdot }^{C}$, ${\bar{p}}_{\cdot j}^{C}$, and ${\bar{p}}^{C}$ denote the means over *j*, *i*, and both *i* and *j*, respectively. With these means, the distances can be doubly centered by

(10) $$q_{ij}^{C}=p_{ij}^{C}-{\bar{p}}_{i\cdot }^{C}-{\bar{p}}_{\cdot j}^{C}+{\bar{p}}^{C},$$

and likewise for $q_{ij}^{S}$. DC between $G^{C}$ and $G^{S}$ is given by

(11) $$\mathrm{DC}\left(G^{C},G^{S}\right)=\frac{\mathrm{dCov}(G^{C},G^{S})}{\sqrt{\mathrm{dCov}\left(G^{C},G^{C}\right)\mathrm{dCov}\left(G^{S},G^{S}\right)}},$$

where

(12) $$\mathrm{dCov}\left(G^{C},G^{S}\right)=\frac{1}{N}\sqrt{\sum_{i}\sum_{j}q_{ij}^{C}q_{ij}^{S}}.$$

$\mathrm{dCov}(G^{C},G^{C})$ and $\mathrm{dCov}(G^{S},G^{S})$ are defined likewise. DC can be computed for arbitrary matrices with *N* columns. DC is in [0,1], and a lower value means $G^{C}$ and $G^{S}$ are less correlated. We aim at DC being close to 0.

#### 5.5.1. Baselines

To compute the lower bound DC on the WikiArt test dataset, we assigned the one-hot vector of the ground-truth genre and style movement labels as the content and style embeddings, representing the uppermost disentanglement when the labels are 100% correct. Besides the lower bound, we evaluated DC on ResNet50 [61], CLIP [14], and DINO [40]. For ResNet50, embeddings were extracted before the last fully connected layer. For CLIP, we used the embedding from the CLIP image encoder $E_{I}$. For pre-trained DINO, following Splice [39], content and style embeddings were extracted from the deepest layer from the self-similarity of keys in the attention module and the [CLS] token, respectively.

#### 5.5.2. Results

Results are reported in Table 2. With the lowest DC of 0.367, GOYA demonstrates the best disentanglement, surpassing the second-best model fine-tuned CLIP by a large margin. With only nearly 1/3 training parameters of ResNet50 and 1/20 of CLIP, GOYA outperforms embeddings directly trained on WikiArt’s real paintings while consuming fewer resources. Also, GOYA achieves better disentanglement capability than DINO, with much more compact embeddings, e.g., 1/300 content size embedding. However, there is still a notorious gap between GOYA and the lower bound based on labels, showing that there is room for improvement.

### 5.6. Similarity Retrieval

Next, we evaluate the visual retrieval performance of GOYA. Given a painting as a query, the five closest images are retrieved based on the cosine similarity of the embeddings in the content and style space, representing the most similar paintings in each space.

#### Results

**Figure 4:**
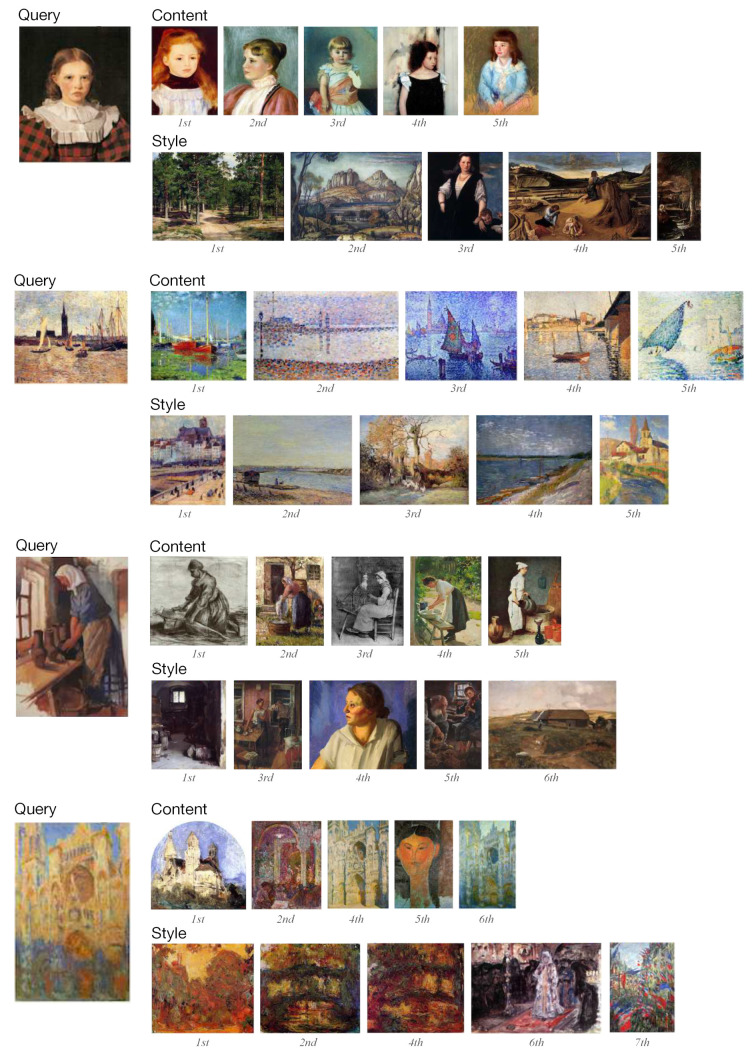
Similarity retrieval in the content and style spaces using GOYA on the WikiArt test set. The similarity decreases from left to right. Copyrighted images are skipped.

Visual results are shown in Figure 4. Most of the paintings retrieved in the content space depict scenes similar to the query image. For instance, in the third query image, a woman with a headscarf is depicted bending over to scrub a pot, while all similar paintings in the content space show a woman leaning to do manual labor such as washing, knitting, and chopping, independently of their visual style. It can be seen that, in most similar content paintings, various styles are depicted through different color compositions and tones. On the contrary, similar paintings in the style space tend to exhibit similar styles but different content. Similar images in the style space possess similar color compositions or brushstrokes, but depict distinct scenes compared to the query image. For example, the fourth query image, one of the paintings in the “*Rouen Cathedral*” series by Monet, exhibits different visual appearances on the same object under the light variance. It can be observed that the retrieved images in the style space also employ different light conditions to create a sense of space and display vivid color contrast. Furthermore, they also display similar color compositions and strokes but paint different scenes. More results can be found in the Appendix A.

### 5.7. Classification Evaluation

For evaluating the disentangled embeddings for art classification, following the protocol in [66], we trained two independent classifiers with a single linear layer on top of the content and style embeddings. We used 10 genres (genre labels include *abstract painting, cityscape, genre painting, illustration, landscape, nude painting, portrait, sketch and study, religious painting,* and *still life*) and 27 style movements (style movement labels include *Abstract Expressionism, Action painting, Analytical Cubism, Art Nouveau, Baroque, Color Field Painting, Contemporary Realism, Cubism, Early Renaissance, Expressionism, Fauvism, High Renaissance, Impressionism, Mannerism Late Renaissance, Minimalism, Naive Art Primitivism, New Realism, Northern Renaissance, Pointillism, Pop Art, Post Impressionism, Realism, Rococo, Romanticism, Symbolism, Synthetic Cubism* and *Ukiyo-e*) in the WikiArt [9] dataset for classification evaluation.

#### 5.7.1. Baselines

We compared GOYA against three types of baselines: pre-trained models, models trained on WikiArt dataset, and models trained on diffusion generated images. As pre-trained models, we used the Gram matrix [59,67], ResNet50 [61], CLIP [14], and DINO [40]. For models trained on WikiArt, other than fine-tuning ResNet50 and CLIP, we also applied two popular contrastive learning methods: SimCLR [60] and SimSiam [66]. For models trained on generated images, ResNet50 and CLIP are fine-tuned with style movements in the prompts. When fine-tuning ResNet50 and CLIP, a linear classifier was added after the layer where embeddings are extracted, and we then trained the entire model on top of the pre-trained checkpoint. SimCLR and SimSiam were trained without any annotations.

Here we clarify the layer where the embeddings were extracted. Gram matrix embeddings are computed from the layer *conv5_1* of a pre-trained VGG19 [68]. For ResNet50 [61], CLIP [14], and DINO [40], the protocols for which layer to extract embeddings and for fine-tuning are consistent as in the disentanglement task.

#### 5.7.2. Results

Table 3 shows the classification results. Compared with the pre-trained baselines listed in the first four rows, GOYA surpasses the Gram matrix, ResNet50, and DINO. However, it falls short of the pre-trained CLIP by less than 1% in both genre and style movement accuracy. Compared with models trained on WikiArt, although not comparable to fine-tuned ResNet50 and CLIP on classification, GOYA demonstrates superior disentanglement capabilities, as shown in Table 2. Moreover, GOYA exhibits enhanced classification performance when compared to contrastive learning models SimCLR and SimSiam.

When trained on diffusion generated images, GOYA achieves the best classification performance compared to other models with different embedding sizes. After fine-tuning on style movement in the prompts, ResNet50 shows a 3% increase on the style accuracy, indicating the potential for analysis via synthetically generated images. However, CLIP decreases in both genre and style accuracy after fine-tuning on generated images. SimCLR experiences a dramatic decrement when trained on generated images compared to WikiArt. As SimCLR focuses more on learning the intricacies of the image itself rather than the relation of images, it learns the distribution of generated images, leading to poor performance on WikiArt. While training on the same dataset, GOYA maintains better capability on classification tasks while achieving high disentanglement.

**Figure 5:**
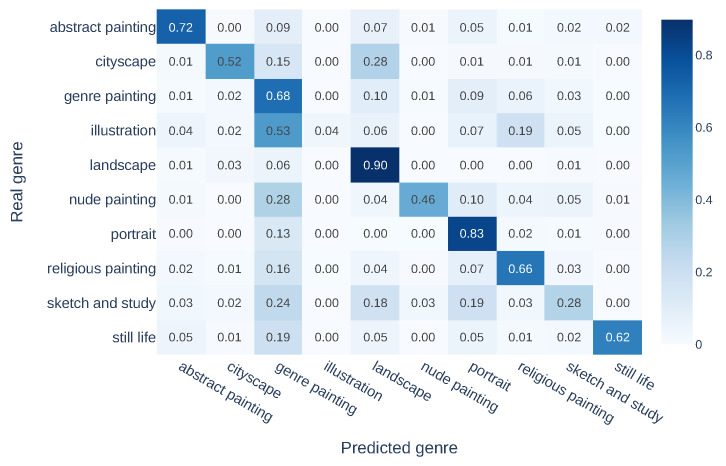
Confusion matrix for genre classification evaluation in the content space using GOYA.

To thoroughly examine the classification results, we provide confusion matrix analyses for both genre and style movement classification evaluations. Figure 5 shows the confusion matrix of genre classification evaluation on GOYA’s content space. The number in each cell represents the proportion of images classified as the predicted label to the total images with the true label. The darker the color, the more images are classified as the predicted label. We can observe that images from several genres are misclassified as *genre painting*, as such paintings usually depict a wide range of activities in daily life, thus overlapping semantically with images from other genres, such as *illustration* and *nude painting*. In addition, due to the high similarity of depicted scenes, there is a 28% misclassification rate of images from *cityscape* as *landscape*.

**Figure 6:**
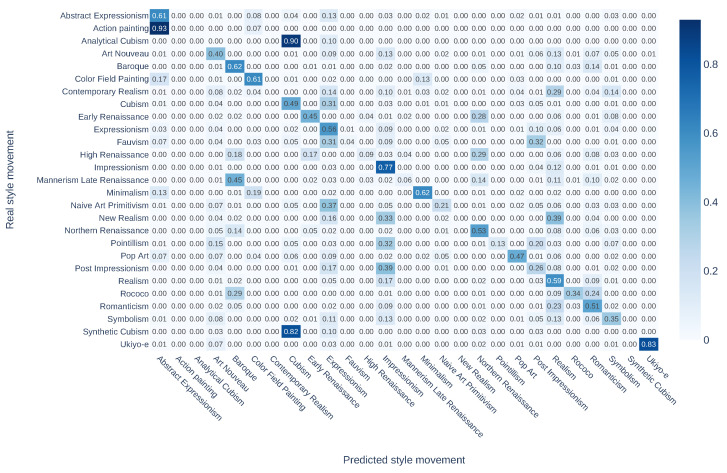
Confusion matrix for style movement classification evaluation in the style space using GOYA.

The confusion matrix of style movement classification is shown in Figure 6. However, the boundary of some movements is not very clear, as some movements are sub-movements that represent different phases within one major movement, e.g., *Synthetic Cubism* in *Cubism* and *Post Impressionism* in *Impressionism*. Generative models may produce images likely to belong to the major movement even when the prompt is about sub-movements, leading GOYA to learn from inaccurate information. Thus, images from sub-movements are prone to be predicted as the according major movement. For example, 82% of the images in *Synthetic Cubism* and 90% of the images in *Analytical Cubism* are classified as *Cubism*. Similarly, about 1/3 of the images in *Contemporary Realism* and *New Realism* are predicted incorrectly as *Realism*.

### 5.8. Ablation Study

We conducted an ablation study on the WikiArt test set to assess the effectiveness of the losses and the network structure in GOYA.

#### 5.8.1. Losses

We compare the losses used in GOYA against two other popular contrastive losses, Triplet loss [69] and NTXent loss [70], both of which have shown their superiority in many contrastive learning methods. We also investigated the application of a style classification loss in conjunction with the above-mentioned contrastive losses. The criteria of selecting positive and negative pairs remain consistent across all of these loss functions.

**Figure 7:**
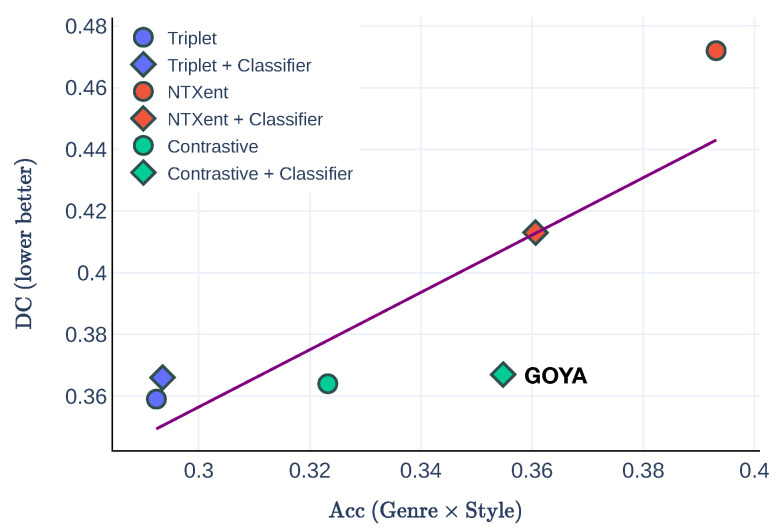
Loss comparison. The *x*-axis shows the product of genre and style accuracies (the higher the better), while the *y*-axis presents the disentanglement, DC (the lower the better). The purple line shows the trendline as y=0.0776+0.9295x. In general, better accuracy is obtained at the expense of a worse disentanglement. Only GOYA (Contrastive + Classifier loss) improves accuracy without damaging DC.

The results in terms of accuracy (as the product of genre and style movement accuracies) and disentanglement (as DC) are depicted in Figure 7. The NTXent loss achieves the highest accuracy but with the cost of undercutting the disentanglement ability. In contrast, Triplet loss exhibits almost the best disentanglement performance but lags behind in terms of classification performance. Compared to these two losses, only the contrastive loss in GOYA manages to maintain a balance between disentanglement and classification performance. Moreover, after occupying the classification loss, GOYA has a boost in classification accuracy without sacrificing disentanglement, achieving the best performance compared to the other loss settings.

#### 5.8.2. Embedding Size

**Figure 8:**
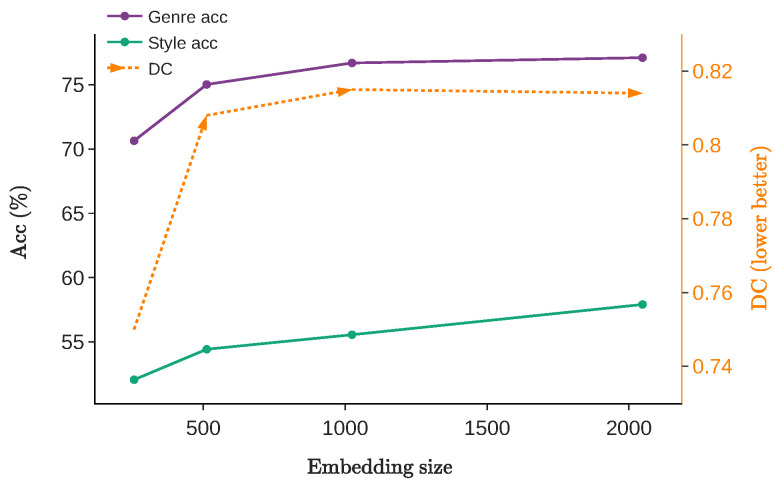
Disentanglement and classification evaluation with different embedding sizes when only one single layer is set in the content and style encoder. A larger embedding size benefits the genre and style movement accuracy but leads to worse disentanglement.

We explore the effect of the embedding size on a single-layer content and style encoders, ranging from 256 to 2048. Figure 8 illustrates that both genre and style accuracy improve by up to 6% as the embedding size increases, but conversely, the DC deteriorates, from 0.750 to 0.814, indicating a trade-off between classification and disentanglement. Moreover, the classification performance of genre and style movement surpasses the pre-trained CLIP (shown in Table 3) when the embedding size exceeds 512, suggesting that larger embedding sizes possess a stronger ability to distill knowledge from the pre-trained model. Inspired by this finding, we set the embedding size to 2048.

## 6. Discussion

### 6.1. Image Generation

- **Prompt design**: In this study, we used a combination of content and style descriptions as prompts, where the content description comprises the title of paintings, and the style description employs the style labels of the WikiArt dataset. Alternatively, more specialized prompt designs could be implemented to attain even finer control over the generated images. For example, captions from vision-language datasets could be employed as content descriptions, while detailed style descriptions could be extracted from external knowledge such as Wikipedia.
- **Data replication**: As demonstrated in previous research [71,72], Stable Diffusion might produce forgeries, generating images that closely resemble the training data. However, the extent of these replicated images within our training data remains uncertain, and their potential impact on model training has yet to be thoroughly explored.

### 6.2. Model Training

**Figure 9:**
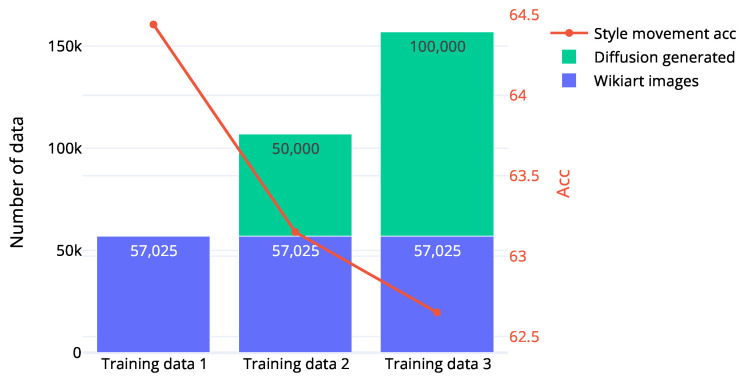
Style classification on ResNet50 when the training set contains both synthetic and real data. As the partition of synthetic images increases, the style movement accuracy drops.

- **Encoder structure**: For the content and style encoders, we employ small networks consisting of only two and three layers, respectively. We found that a higher-dimensional hidden layer (2048) and fewer layers (3) are effective for learning content embeddings, while a lower-dimensional hidden layer (512) and more layers (2) yield better style embeddings. We hypothesize that content embedding, which reflects semantic information, benefits from a large number of neutrons, while style embedding, containing low-level features, is more efficiently represented with lower dimensions.
- **Partition of synthetic images**: We performed style movement classification on a training dataset comprising both synthetic and real data. Results presented in Figure 9 indicate that, as the number of synthetic images increases during training, the accuracy decreases. We attribute this phenomenon to the domain gap between synthetic and real images. In addition, we suggest that contrastive learning may help alleviate the impact of this domain gap.

### 6.3. Limitation on the WikiArt Dataset

While the WikiArt dataset serves as our evaluation dataset, it comes with limitations related to annotations and diversity. Firstly, the annotated genre and style movement label may not entirely align with the content and style definitions described in this paper. Secondly, the majority of the paintings in WikiArt belong to Western art, especially European and American art, thus lacking representation from a diverse spectrum of art paintings. Future work could focus on obtaining more precise annotations for content and style in paintings, as well as including art paintings from various regions, such as Asian, Oceanian, and African art, thereby enriching the diversity of the dataset.

### 6.4. Applications

- **Art applications**: Our work can potentially be extended into various practical scenarios. For instance, it could be integrated into an art retrieval system, enabling users to find paintings based on text descriptions or a given artwork. Additionally, it could be employed in a painting recommendation system, offering personalized suggestions to users according to their preferred paintings. These applications have the potential to enhance user experience and engagement, thus contributing to the improvement of art production and consumption.
- **Digital humanities**: While our work mainly focuses on the analysis of fine art, there is potential for our work to be applied in other areas within digital humanities, such as graphic design and historical document analysis.
- **Beyond art**: Apart from the art domain, audio disentanglement could be a potential area to expand [73,74,75].

## 7. Conclusions

This work proposes GOYA, a method for disentangling content and style embeddings of paintings by training on synthetic images generated with Stable Diffusion. Exploiting the multi-modal CLIP latent space, we first extracted off-the-shelf embeddings to then learn similarities and dissimilarities in content and style with two encoders trained with contrastive learning. Evaluation on the WikiArt dataset included disentanglement, classification, and similarity retrieval. Despite relying only on synthetic images, results showed that GOYA achieves good disentanglement between content and style embeddings. This work sheds light on the adoption of generative models in the analysis of the digital humanities.

## Figures and Tables

**Table 1:** GOYA detailed architecture.

Components	Layer Details
Content encoder C	Linear layer(512,2048), ReLU, Linear layer(2048,2048)
Style encoder S	Linear layer(512,512), ReLU, Linear layer(512,512), ReLU, Linear layer(512,2048)
Projector $h^{C}/h^{S}$	Linear layer(2048,2048), ReLU, Linear layer(2048,64)
Style classifier R	Linear layer(2048,27)

**Table 2:** Distance Correlation (DC) between content and style embeddings on the WikiArt test set. *Labels* indicate the results when using a one-hot vector embedding of the ground truth labels. ResNet50 and CLIP are fine-tuned on WikiArt, while DINO loads the pre-trained weights. The bold font highlights the best results.

Model	Training	Training	Emb. Size	Emb. Size	DC ↓
	Params	Data	Content	Style	
*Labels*	*-*	*-*	*27*	*27*	*0.269*
ResNet50 [61]	47M	WikiArt	2048	204	0.635
CLIP [14]	302M	WikiArt	512	512	0.460
DINO [40]	−	−	616,225	768	0.518
GOYA (Ours)	15M	Diffusion	2048	2048	**0.367**

**Table 3:** Genre and style movement accuracy on the WikiArt [9] dataset for different models. The bold font highlights the best results.

Model	Training	Label	Num.	Emb. Size	Emb. Size	Accuracy	Accuracy
	Data		Train	Content	Style	Genre	Style
Pre-trained							
Gram Matrix [59,67]	-	-	-	4096	4096	61.81	40.79
ResNet50 [61]	-	-	-	2048	2048	67.85	43.15
CLIP [14]	-	-	-	512	512	71.56	51.23
DINO [40]	-	-	-	616,225	768	51.13	38.81
Trained on WikiArt							
ResNet50 [61] (Genre)	WikiArt	Genre	57,025	2048	2048	79.13	43.17
ResNet50 [61] (Style)	WikiArt	Style	57,025	2048	2048	67.22	64.44
CLIP [14] (Genre)	WikiArt	Genre	57,025	512	512	80.43	34.98
CLIP [14] (Style)	WikiArt	Style	57,025	512	512	56.28	63.02
SimCLR [60]	WikiArt	-	57,025	2048	2048	65.82	45.15
SimSiam [66]	WikiArt	-	57,025	2048	2048	51.65	31.24
Trained on Diffusion generated							
ResNet50 [61] (Movement)	Diffusion	Movement	981,225	2048	2048	61.78	45.79
CLIP [14] (Movement)	Diffusion	Movement	981,225	512	512	52.65	43.58
SimCLR [60]	Diffusion	-	981,225	2048	2048	33.82	20.88
GOYA (Ours)	Diffusion	-	981,225	2048	2048	69.70	50.90

**Figure A1:**
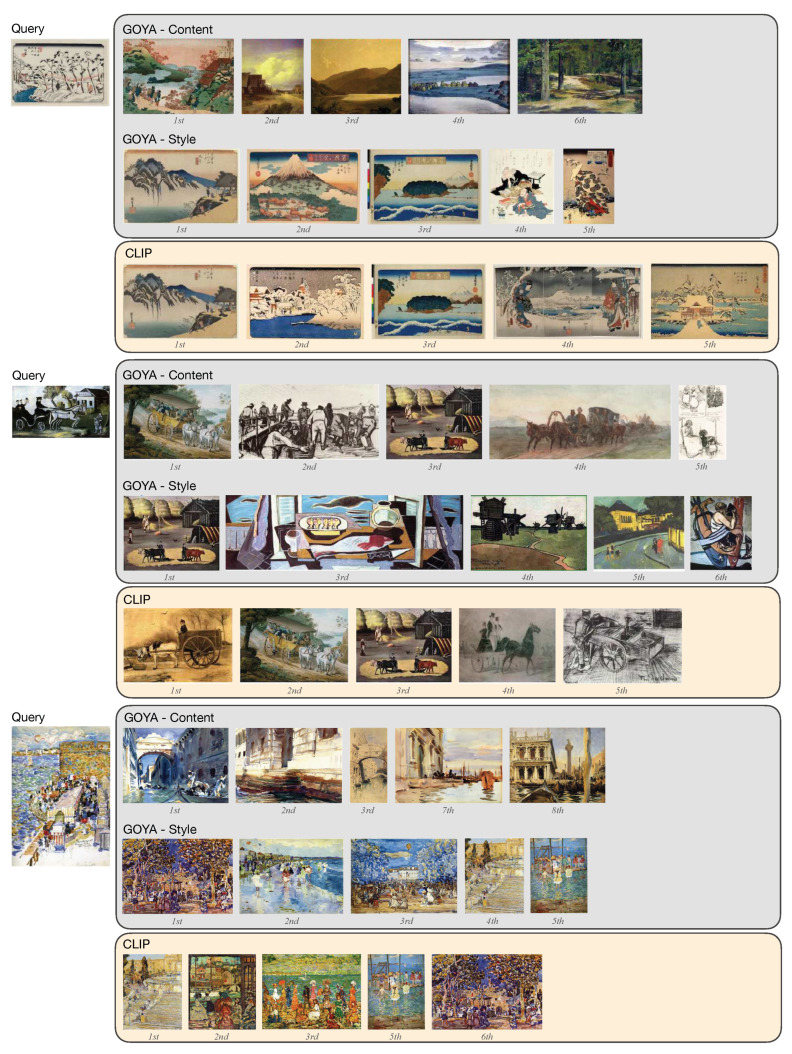
Retrieval results in GOYA content and style spaces and CLIP latent space based on cosine similarity. In each row, the similarity decreases from left to right. Copyrighted images are skipped.

**Figure A2:**
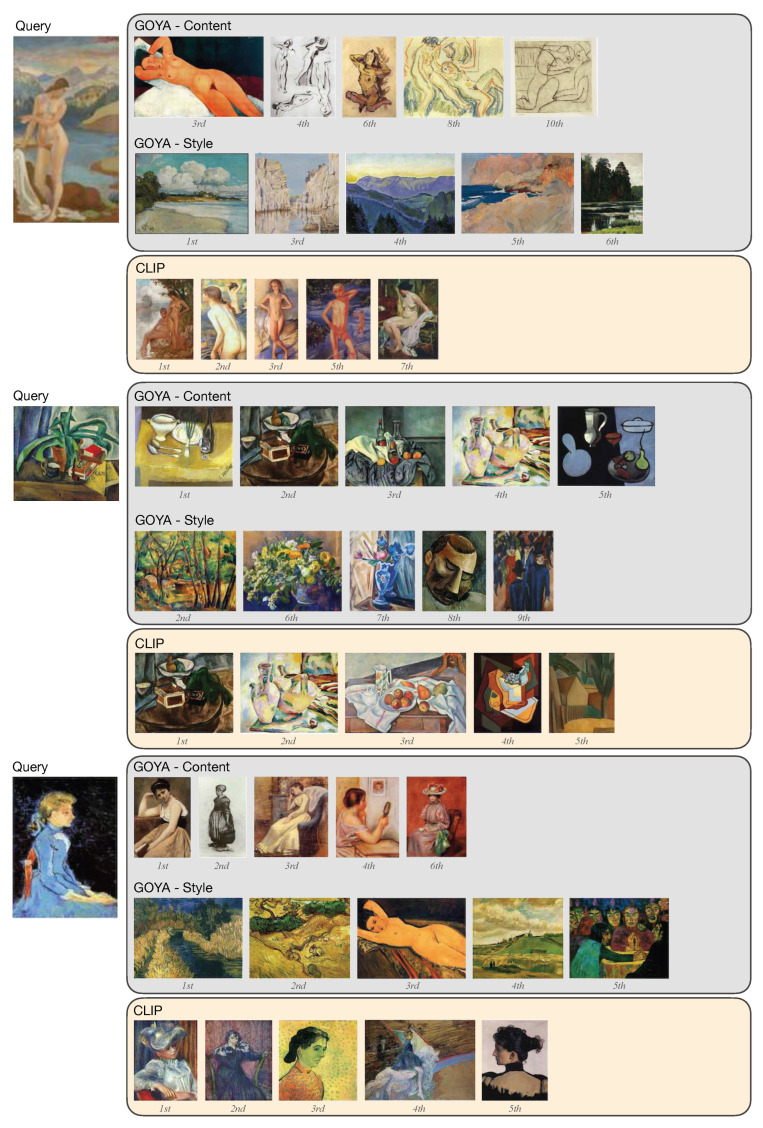
Retrieval results in GOYA content and style spaces and CLIP latent space based on cosine similarity. In each row, the similarity decreases from left to right. Copyrighted images are skipped.

## Data Availability

All images presented in the paper are from the WikiArt dataset or generated by Stable Diffusion. The copyright status of all the images presented in the paper is public domain.

## Appendix A. Similarity Retrieval

Figure A1 and Figure A2 show results comparing against CLIP. In Figure A1 and Figure A2, for each query image, the first two rows display the retrieved images from GOYA content and style spaces, and the last row shows images retrieved in the CLIP latent space. Results show that images in the CLIP latent space are similar in content and style, while in GOYA content space, there is consistency in depicting scenes but with different styles, and in GOYA style space, the visual appearance is similar, but the content is different.
